# The Örebro Musculoskeletal Pain Screening Questionnaire-Short Form and 2-year follow-up of registered work disability

**DOI:** 10.1093/eurpub/ckad079

**Published:** 2023-05-16

**Authors:** Eveliina Heikkala, Petteri Oura, Olli Ruokolainen, Leena Ala-Mursula, Steven J Linton, Jaro Karppinen

**Affiliations:** Research Unit of Population Health, University of Oulu, Oulu, Finland; Medical Research Center Oulu, University of Oulu and Oulu University Hospital, Oulu, Finland; Wellbeing Services, County of Lapland, Rovaniemi, Finland; Medical Research Center Oulu, University of Oulu and Oulu University Hospital, Oulu, Finland; Research Unit of Health Sciences and Technology, University of Oulu, Oulu, Finland; Research Unit of Population Health, University of Oulu, Oulu, Finland; Medical Research Center Oulu, University of Oulu and Oulu University Hospital, Oulu, Finland; Research Unit of Population Health, University of Oulu, Oulu, Finland; Clinical Psychology, Center for Health and Medical Psychology, Örebro University, Örebro, Sweden; Medical Research Center Oulu, University of Oulu and Oulu University Hospital, Oulu, Finland; Research Unit of Health Sciences and Technology, University of Oulu, Oulu, Finland; Finnish Institute of Occupational Health, Oulu, Finland; Rehabilitation Services of South Karelia Social and Health Care District, Lappeenranta, Finland

## Abstract

**Background:**

The Örebro Musculoskeletal Pain Screening Questionnaire (ÖMPSQ) was developed to identify psychological and functioning-related risk factors among individuals with musculoskeletal pain at risk of work disability. This study aimed to examine whether the short version of the ÖMPSQ (ÖMPSQ-SF) can be used for this purpose, using registry-based outcomes.

**Methods:**

The ÖMPSQ-SF was completed by the members of the Northern Finland Birth Cohort 1966 at the age of 46 years (baseline). These data were enriched with national registers, including information on sick leaves and disability pensions (indicators of work disability). The associations between the ÖMPSQ-SF categories (low-, medium- and high risk) and work disability over a 2-year follow-up were analysed using negative binomial regression and binary logistic regression models. We made adjustments for sex, baseline education level, weight status and smoking.

**Results:**

Overall, 4063 participants provided full data. Of these, 90% belonged to the low-risk, 7% to the medium-risk and 3% to the high-risk group. Compared to the low-risk group, the high-risk group had a 7.5 [Wald 95% confidence interval (CI) 6.2–9.0] times higher number of sick leave days and 16.1 (95% CI 7.1–36.8) times higher odds of disability pension after adjustments in the 2-year follow-up.

**Conclusions:**

: Our study suggests that the ÖMPSQ-SF could be used for predicting registry-based work disability at midlife. Those allocated to the high-risk group seemed to have a particularly great need of early interventions to support their work ability.

## Introduction

Persistent musculoskeletal (MSK) pain affects a great number of people worldwide.^1^^,^^2^ It is one of the main reasons that people experience work disability and seek healthcare, and thus causes major economic costs for societies.^2–4^ Particularly back pain and osteoarthritis are among the leading causes for MSK pain-related work disability.^5^ The prognosis of persistent MSK pain is relatively poor^6^ and treatment challenging, often requiring efforts from a variety of healthcare professionals.^2^ Early identification of individuals at risk of persistent MSK pain is therefore crucial to prevent the development of this undesirable outcome and alleviate the related society- and individual-level burden.

Several psychological factors, such as fear-avoidance behaviour, pain catastrophizing, depression, anxiety, and stress, have been recognized as influencing the prognosis of MSK and resulting in poorer pain outcomes, including disability^7^ and increased use of healthcare services.^8^ It has been suggested that fear-avoidance beliefs and psychological stress may mediate the relationship between pain and disability.^9^^,^^10^ A recent high-quality systematic review and meta-analysis showed that rehabilitation interventions that encompass psychological elements are more effective than traditional ones that do not include these entities.^11^ These findings stress the importance of adequate and early screening of psychological factors among people with MSK pain. In recent years, multiple tools have been developed for this purpose. One of these is the validated Örebro Musculoskeletal Pain Screening Questionnaire (ÖMPSQ),^12–14^ which is proposed to predict persistent pain, perceived mental and physical health, disability in daily activities^12^^,^^13^^,^^15^^,^^16^ and importantly, work disability.^12^^,^^15–18^ Developed by Linton *et al.*,^12^ the ÖMPSQ includes 25 items assessing e.g. symptom-, function- and work-related factors (such as pain duration and intensity and thoughts about being able to work in future) in addition to psychological elements (such as fear-avoidance and feelings of depression and anxiety).^14^ Its construct validity and predictive validity have been confirmed in various settings and populations.^12–16^

To date, most previous ÖMPSQ studies have used the full version of the questionnaire in their analyses. However, a decade ago, a short version, the 10-item ÖMPSQ (ÖMPSQ-SF), was developed to meet the temporal challenges related to clinical work. The ÖMPSQ-SF correlates highly with the full version^19^ and has comparable properties to the ÖMPSQ in the prediction of work-related factors in particular (e.g. absenteeism, sick leave and return to work).^19–21^ Still, there are some limitations to the implementation of the ÖMPSQ-SF, such as a lack of registry-based work disability outcome data with large study samples and over 1-year follow-ups. If the individuals at the highest risk of long-term work disability could be accurately identified from among the vast amount of people with MSK pain,^22^ interventions aimed at preventing work disability could be designed in a more targeted manner and utilized at the earliest convenience.

The aim of the present population-based birth cohort study was to investigate whether the results of the ÖMPSQ-SF are associated with registry-based work disability, defined as sick leave days and disability pension, in a 2-year follow-up among middle-aged Finns. Our hypothesis was that higher ÖMPSQ-SF risk scores (i.e. a high-risk group), in comparison to the lowest ones (i.e. a low-risk group), is significantly related to both work disability outcomes.

## Methods

### Study population

The study population belonged to the Northern Finland Birth Cohort 1966 (NFBC1966),^23^^,^^24^ which initially comprised pregnant women with an expected date of delivery in 1966 and who lived in the two northernmost provinces of Finland. A total of 12 231 children belong to the NFBC1966, covering 96.3% of all births in the area in 1966. Since their mothers’ antenatal clinic visit, the NFBC1966 members have been followed longitudinally by repeated data collections. During the latest data collection in 2012–14, when the NFB1966 members were ∼46 years old, those who were alive and whose addresses were known were contacted via electronic/postal questionnaires and invited to participate in a clinical examination. Questionnaire data were received from 7146 participants (69% of the target population of 10 331) and clinical examination data from 5832 participants (56%). We asked the participants to fill in the ÖMPSQ-SF only if they had had any MSK pain during the last 12 months (*n* = 4704, 46%). The questionnaire- and health examination-based data were then linked to register-based 2-year follow-up data on the sick leave days and disability pensions of those who had given written permission for this process. The participants who had been granted disability pension before the baseline were excluded. Finally, complete data and the required written consent to use the data were available for 4063 participants (39%). The study was approved by the Northern Ostrobothnia Hospital District Ethical Committee 94/2011 (12 December 2011).

### Sick leave days and disability pensions (outcomes)

The information on sick leave days and disability pensions was gathered from the national registers of The Social Insurance Institution of Finland (SII) and the Finnish Centre for Pensions. The follow-up period was 2 years (730 days) for each participant, starting at the date on which they completed the ÖMPSQ-SF. If the date was not known (a participant had not reported it), the starting date was primarily the date of the clinical health examination and secondarily the population’s average date of completing the ÖMPSQ-SF.

The total number of sick leave days during the follow-up was calculated and considered a count variable. It is important to note that the SII does not register sick leave periods shorter than 10 weekdays: these are paid by employers, with few exceptions among entrepreneurs, and therefore, our data depicted the number of sick leave days from sick leave periods that had lasted longer than 10 weekdays. In general, the SII pays sickness allowances for 300 weekdays, after which eligibility for disability pension is considered either fixed-term (called rehabilitation subsidy) or permanent.

With respect to long-term work disability, the number of days for which a participant had received any type of long-term disability allowance (full- or part-time, fixed-term or permanent disability pension) within the 2-year follow-up was evaluated and dichotomized as zero (‘no’) vs. one or more (‘yes’).

### 10-item Örebro Musculoskeletal Pain Screening Questionnaire (exposure)

A Finnish translation of the short version of the validated ÖMPSQ,^14^ the ÖMPSQ-SF, contains 10 items on: (i) the duration of pain(s), (ii) pain rating, (iii) the ability to do light work, (iv) the ability to sleep at night, (v) feelings of anxiety, (vi) feelings of depression, (vii) the perceived risk of pain becoming chronic, (viii) opportunities to return to work and (ix and x) fear-avoidance beliefs. Each item was scored 0–10, 0 referring to absence of impairment and 10 to severe impairment. The scores were then summed up and the respondents were divided into three groups according to their total score: (i) low risk (0–40 points); (ii) medium risk (41–50 points); and (iii) high risk (51–100 points).^16^ The validity of the original^19^ and several translated versions of the ÖMPSQ-SF has been previously documented.^25–27^

### Confounders

We accounted for the potentially confounding effects of the following on the association between the ÖMPSQ-SF risk groups, sick leave days and disability pension: sex, education level, weight status and smoking at baseline.^28^^,^^29^ Data on these confounders were collected from birth records, questionnaires and health examination at 46 years (baseline).

Information on sex (female/male) was gathered from birth records. Education level was divided into three categories according to school years accumulated until the age of 46: (i) compulsory or no basic education (≤9 years), (ii) secondary (10–12 years) and (iii) tertiary (>12 years). Height and weight were based on measurements taken during the health examination and were converted into BMI (kg/m^2^), which was used for indicating the participants’ weight status. The following cut-off points were utilized: normal weight ≤24.99 kg/m^2^, overweight 25.00–29.99 kg/m^2^ and obesity ≥30.00 kg/m^2^. Participants were categorized into ‘non-smokers’, ‘former smokers’ and ‘current smokers’ on the basis of their responses to the questions: ‘Have you ever smoked regularly?’ and ‘Do you currently smoke?’.

### Statistical methods

The demographic characteristics of the study sample were described using numbers and percentages for categorical variables and medians and interquartile ranges (IQRs) for continuous variables due to the non-normal distribution of the data. The statistical significance in these analyses was estimated using the Chi-square and Kruskal–Wallis tests, with a *P*-value of <0.05 being deemed statistically significant.

Negative binomial regression analysis with a log link was used to study the association between the ÖMSPQ-SF risk groups (exposure) and sick leave days (outcome), and these results were presented as incidence rate ratios (IRRs) and their 95% Wald confidence intervals (CIs). IRR above and below 1 indicated a higher and lower incidence of the outcome in the explored group than in the reference group, respectively. Binary logistic regression analysis was carried out to calculate odds ratios (ORs) and 95% CIs for the association between the ÖMSPQ-SF risk groups (exposure) and any type of new disability pension (outcome). Both analyses included unadjusted and adjusted models (including sex, education level, weight status and smoking as confounders). The low-risk group according to the ÖMSPQ-SF was assessed as the reference. SPSS versions 25 and 27 were used for the analyses.

## Results

Table 1 presents the characteristics of the study sample (*n* = 4063). Most of the participants were female (58%), had secondary education (66%), were of normal weight or overweight (39% and 40%, respectively), and did not smoke at baseline (current smoking: 17%). Altogether, 90% of the participants belonged to the low-risk group, 7% to the medium-risk group and 3% to the high-risk group, according to the ÖMPSQ-SF.

**Table 1:** Characteristics of participants, stratified by ÖMPSQ-SF groups

	**Total** **(*n* = 4063)**	**Low risk** **(*n* = 3646)**	**Medium risk** **(*n* = 293)**	**High risk** **(*n* = 124)**	***P*-value** **(Chi-square/Kruskal–Wallis test)**
Sex, % (*n*)					0.061
Females	58 (2365)	58 (2102)	61 (180)	67 (83)	
Males	42 (1698)	42 (1544)	39 (113)	33 (41)	
Education level, % (*n*)					<0.001
Compulsory or no basic	5 (208)	5 (171)	7 (21)	13 (16)	
Secondary	66 (2687)	65 (2,393)	73 (215)	64 (79)	
Tertiary	29 (1168)	30 (1082)	20 (57)	23 (29)	
Weight status, % (*n*)					<0.001
Normal	39 (1598)	40 (1463)	30 (86)	39 (49)	
Overweight	40 (1624)	40 (1459)	43 (127)	31 (38)	
Obesity	21 (841)	20 (724)	27 (80)	30 (37)	
Smoking, % (*n*)					<0.001
Non-smoker	55 (2237)	56 (2052)	45 (130)	44 (55)	
Former smoker	28 (1121)	28 (1010)	28 (83)	23 (28)	
Current smoker	17 (705)	16 (584)	27 (80)	33 (41)	
Number of days on sick leave (longer than 2 weeks)^a^					<0.001
Median (IQR)	0 (0–0)	0 (0–0)	0 (0–23)	2 (0–84)	
Occurrence of new registered disability pension, % (*n*)^a^					<0.001
Yes	1 (36)	1 (15)	3 (10)	9 (11)	
No	99 (4027)	99 (3631)	97 (283)	91 (113)	

ÖMPSQ-SF, Short form of Örebro Musculoskeletal Pain Screening Questionnaire; IQR, interquartile range.

a During 2-year follow-up, starting when ÖMPSQ-SF was filled in.

The participants in the high-risk group were most likely to have completed compulsory education or to have no basic education, be obese and currently smoke [e.g. the prevalence of compulsory or no basic education: 13% (high risk), 7% (medium risk) and 5% (low risk)] (*P* < 0.001 for all) (table 1). The median number of sick leave days during the 2-year follow-up was two (IQR: 0–84) in the high-risk group, compared with 0 in the medium- and low-risk groups (IQR: 0–23 and 0–0, respectively) (*P* < 0.001). Similarly, a higher percentage of participants in the high-risk group than in the medium- and low-risk groups had been granted disability pension during the follow-up (9% vs. 3% and 1%, respectively) (*P* < 0.001).

The participants who belonged to the high-risk group had accumulated a 7.5-fold higher number of sick leave days during the 2-year follow-up than the low-risk group participants [adjusted IRR 7.5 (95% Wald CI 6.2–9.0)] (table 2). They also had 16.1-fold higher odds of disability pension after all confounders (sex, education level, weight status and smoking at baseline) were controlled for (adjusted OR 16.1, 95% CI 7.1–36.8) (table 3). As for the medium-risk group, we detected significant associations with sick leave days and disability pension, but they were weaker than those of the high-risk group (sick leave days: adjusted IRR 3.7, 95% Wald CI 3.3–4.2; disability pension: adjusted OR 6.8, 95% CI 3.0–15.7) (tables 2 and 3).

**Table 2:** Association between ÖMPSQ-SF groups and number of days on sick leave (longer than 2 weeks) at 2-year follow-up in midlife

ÖMPSQ-SF groups	*N*	IRR (95% Wald CI)
Unadjusted		
Low risk	3646	Ref.
Medium risk	293	4.3 (3.8–4.8)
High risk	124	8.2 (6.9–9.9)
Adjusted^a^		
Low risk	3646	Ref.
Medium risk	293	3.7 (3.3–4.2)
High risk	124	7.5 (6.2–9.0)

ÖMPSQ-SF, Short form of Örebro Musculoskeletal Pain Screening Questionnaire; IRR, incidence rate ratio; CI, confidence interval.

a Adjusted for sex, education level, weight status and smoking at baseline.

**Table 3:** Association between ÖMPSQ-SF groups and occurrence of new registered disability pension at 2-year follow-up in midlife, presented as ORs and their 95% CIs

ÖMPSQ-SF groups	*N*	Disability pension	*N*	No disability pension
Unadjusted				
Low risk	15	Ref.	3631	
Medium risk	10	8.6 (3.8–19.2)	283	Ref.
High risk	11	23.6 (10.6–52.5)	113	Ref.
Adjusted^a^				
Low risk	15	Ref.	3631	
Medium risk	10	6.8 (3.0–15.7)	283	Ref.
High risk	11	16.1 (7.1–36.8)	113	Ref.

ÖMPSQ-SF, Short form of the Örebro Musculoskeletal Pain Screening Questionnaire; OR, odds ratio; CI, confidence interval.

a Adjusted for sex, education level, weight status and smoking at baseline.

## Discussion

Prior research has shown that screening questionnaires, such as the ÖMPSQ, may help identify individuals at risk of work disability.^12^^,^^15–18^ This large population-based study found that the high-risk group, according to the ÖMPSQ-SF, had a 7.5-fold higher number of sick leave days and 16-fold higher odds of disability pension than the low-risk group during a 2-year follow-up. Significant associations between the medium-risk group and these registry-based outcomes were also observed, but they were weaker than those of the high-risk group.

At baseline, the ÖMPSQ-SF allocated 3% of the participants into the high-risk group. This estimate is far from those of other studies using Brazilian low back pain patients^30^ and Swedish primary care patients^31^ as the study population. Here, we assessed participants who belonged to the birth cohort, and not individuals who had sought medical care, which may explain the differences. There were more females than males in each risk group, but the contrast was the highest in the high-risk group (67%). This finding corresponds to that of Fuhro *et al.*^30^ who also used a categorized ÖMPSQ-SF variable. The high-risk group participants had a tendency to engage in unhealthy behaviour, such as smoking and had overweight/obesity. They have also previously shown to experience severe mental distress (measured by validated questionnaires) more often than the others.^32^ These observations suggest unfavourable health behaviours and mental health burdens among the individuals with high-risk scores in the ÖMPSQ-SF.

The high-risk group had not only a 7.5 times higher number of sick leave days but also 16-fold odds of being granted disability pension during the follow-up, in comparison to the low-risk group. As the individuals who had been granted disability pension before baseline were excluded and several confounders were controlled for, these findings clearly suggest that the ÖMPSQ-SF can be used to predict work disability at midlife. People with high-risk scores in the ÖMPSQ-SF, i.e. experiencing adverse psychosocial and symptom-related factors, may require more targeted, multidisciplinary care with repeated follow-ups than low-risk people, who may be in less need of such measures. Even though the effectiveness of this kind of comprehensive treatment approach has not yet been studied in terms of the ÖMPSQ-SF,^32^ the present results endorse the allocation of limited healthcare resources according to the ÖMPSQ-SF to potentially decrease disability-related economic costs and alleviate the individual-level suffering related to MSK pain and its comorbidities. One relatively simple procedure to be further explored in the future could be motivational interviewing/stratified vocational advice intervention for high-risk individuals.^33^ Finally, the ÖMPSQ-SF is designed to systematize the consideration of psychosocial and functioning-related barriers so that measures to facilitate recovery from MSK problems can be tailored. As these problems are often accompanied by mental health problems, the ÖMPSQ-SF deals with all these leading causes of work disability simultaneously.^34^^,^^35^

Due to the way in which the national registers are constructed, we lacked data on short-term sick leaves and thus were not fully able to capture all sickness absence days. Nevertheless, we believe that prolonged, continuous periods have the most impact at the societal level. Although a great number of people worldwide are affected by MSK pain during their life course,^1^^,^^22^ most of these pains seem to disappear in a few weeks.^36^ Thus, these people may not be the ones whom the ÖMPSQ-SF should primarily identify. In Finland, employers are obligated to give employees sick pay during short-term sick leave episodes (the first 10 weekdays), after which the SII, as a government-funded institute, starts paying sickness allowance. As the registered sickness absence allowances assessed in this study were funded by a government institute, and as such by tax-payers, our results depict notable societal-level impact.

Overall, the present results supplement the existing literature that has associated higher ÖMPSQ-SF scores (high-risk group) with the accumulation of self-reported sick leave days over a 1-year follow-up,^19^ poorer return to work within 3 months,^37^ and prolonged recovery from MSK and soft tissue injuries leading to generally later return to work.^21^^,^^38^ In an Australian 1-year follow-up study,^39^ the ÖMPSQ-SF demonstrated poor discrimination in terms of work absenteeism and presenteeism, but this difference to our findings may originate from the different age groups we studied (young adults vs. middle-aged individuals) and outcome data collection methods (self-reports vs. registry-based). Importantly, in this study, we also clearly showed that not only the high-risk group but also the medium-risk group had a higher registry-based number of sick leave days and likelihood of disability pension. Thus, there is a clear need for further registry- and population-based studies using a trichotomized ÖMPSQ-SF risk group variable to establish the clinical relevance of the medium-risk group. In addition to the ÖMPSQ (and ÖMPSQ-SF), Keele STarT Back Screening Tool is among the most studied. It has shown to have ‘acceptable’ ability to discriminate disability outcomes^18^ and moderate to fair agreement with the ÖMPSQ-SF, but the ÖMPSQ-SF may better identify adverse lifestyle factors.^32^

This study is among the first to show that the ÖMPSQ-SF can also be used to predict registered work disability at midlife among people with MSK pain. Our registry-based outcome variables, longitudinal design and the large population-based dataset are as the main strengths in the current study. In all, the NFBC1966 could be considered generally representative of the middle-aged Finnish population as the participation rate was substantially high at the 46-year data collection point and as there have been observed only minor differences in socioeconomic background between non-participants and participants at baseline of this study.^23^ Moreover, the NFBC1966 members have migrated within the country since their birth.^23^ However, the study also has its limitations. Firstly, only the full version of the ÖMPSQ has been validated in Finland.^14^ Nevertheless, the ÖMPSQ-SF is composed of the subscales of the ÖMPSQ and has passed the validation process in several countries and been constantly documented as a valid instrument.^25–27^ Secondly, while the cut-off of 50 points for the high-risk group was well-established and commonly used,^19^^,^^38^ the cut-offs for the low- and medium-risk groups stemmed from the 25-item ÖMPSQ. Thirdly, individuals on disability pension were excluded from the analyses but those on sick leave at baseline were not. The decision to include these individuals in this study was based on the fact that before receiving grants for disability pension, individuals are generally required to be on sick leave for a year in Finland. Fourthly, owing to the relatively low number of granted disability pensions, some subcategories had a low number of participants, which may have contributed to the accuracy of the estimates in the current results. However, the lower border of the CIs in the disability pension ORs was still high (7.1 in the high-risk group and 3.0 in the medium-risk group), which indicates manifold odds of disability pension among the high-risk and medium-risk participants in contrast to the low-risk participants. Finally, the ÖMPSQ-SF was based on self-reported data, which is generally acknowledged to be susceptible to recall and social desirability bias. However, it should also be noted that some elements, particularly psychological symptoms, cannot be objectively measured.

## Conclusions

The present population-based findings support the use of the ÖMPSQ-SF for predicting registry-based work disability among individuals reporting MSK pain. People allocated into the high-risk group seemed to be in great need of tailored early interventions to prevent work disability. This is an important result in terms of, firstly, the wider implementation of the ÖMPSQ-SF in primary and occupational healthcare settings, and secondly, the allocation of limited healthcare resources in the most cost-effective manner. The wider implementation of the ÖMPSQ-SF to the clinical practise would potentially require more education on the survey not only in primary and occupational healthcare environments but also at medical school. Future research with longer follow-up periods and/or larger sample sizes should be conducted to improve the accuracy of disability pension estimates.

## Data Availability

NFBC data are available from the University of Oulu, Infrastructure for Population Studies. IT is possible to apply for permission to use the data for research purposes via an electronic material request portal. In the use of data, we followed the EU general data protection regulation (679/2016) and the Finnish Data Protection Act. The use of personal data is based on the cohort participant’s written informed consent in their most recent follow-up study, which may cause limitations to its use. Please contact the NFBC project centre (NFBCprojectcenter@oulu.fi) or visit the cohort website (www.oulu.fi/nfbc) for more information.
